# Durable response to sotorasib-based combination therapy in advanced lung adenocarcinoma harboring KRAS G12C and STK11 mutations: a case report

**DOI:** 10.3389/fonc.2026.1776402

**Published:** 2026-03-12

**Authors:** Jiaqi Wang, Yaxin Yan, Quanbing Su, Yitao Jia

**Affiliations:** Department of Oncology, Hebei General Hospital, Shijiazhuang, Hebei, China

**Keywords:** heterogeneous immune phenotype, KRAS G12C, lung adenocarcinoma, sotorasib, STK11

## Abstract

Lung adenocarcinoma is a prevalent and aggressive subtype of non-small cell lung cancer (NSCLC). Mutations in the Kirsten rat sarcoma viral oncogene homologue (KRAS) represent key oncogenic drivers and are associated with poor prognosis. Sotorasib is a KRAS G12C inhibitor. It suppresses tumor growth by specifically and irreversibly locking the mutant KRAS protein. Currently, sotorasib is only approved for later-line treatment of KRAS G12C-mutated NSCLC. Here, we present a case of advanced NSCLC harboring a KRAS G12C and STK11 mutations. The patient received an exploratory first-line therapy with sotorasib combined with immunotherapy and chemotherapy, achieving significant and durable clinical benefit lasting 23 months, with manageable adverse events. This case highlights the potential of sotorasib in combination regimens as a first-line treatment strategy for KRAS G12C-mutated NSCLC and underscores the importance of individualized treatment planning. However, the use of sotorasib in the first-line setting remains an exploratory off-label approach and requires further clinical evidence to validate this treatment strategy.

## Introduction

Lung adenocarcinoma is the most prevalent histological subtype of non-small cell lung cancer (NSCLC). The *KRAS G12C* mutation is a key oncogenic driver, accounting for approximately 1.4-4.3% of NSCLC cases in Asian populations, with approximately 90% of affected patients having a history of smoking (1). Historically considered ‘undruggable’, the *KRAS* pathway has recently become targetable with the development of selective *KRAS G12C* inhibitors, such as sotorasib and adagrasib (2). Sotorasib is an irreversible molecular inhibitor that specifically binds to the *KRAS G12C* mutant protein, locking it in an inactive guanosine diphosphate (GDP)-bound state. Approved by the U.S. Food and Drug Administration (FDA) in May 2021, sotorasib is currently recommended for use as a second-line or later-line treatment in *KRAS G12C*-mutant NSCLC (3). Here, we present a case of advanced lung adenocarcinoma harboring a *KRAS G12C* and *STK11* mutations that achieved disease control with a sotorasib-based combination regimen in the first-line setting.

## Case description

**Figure 1:**
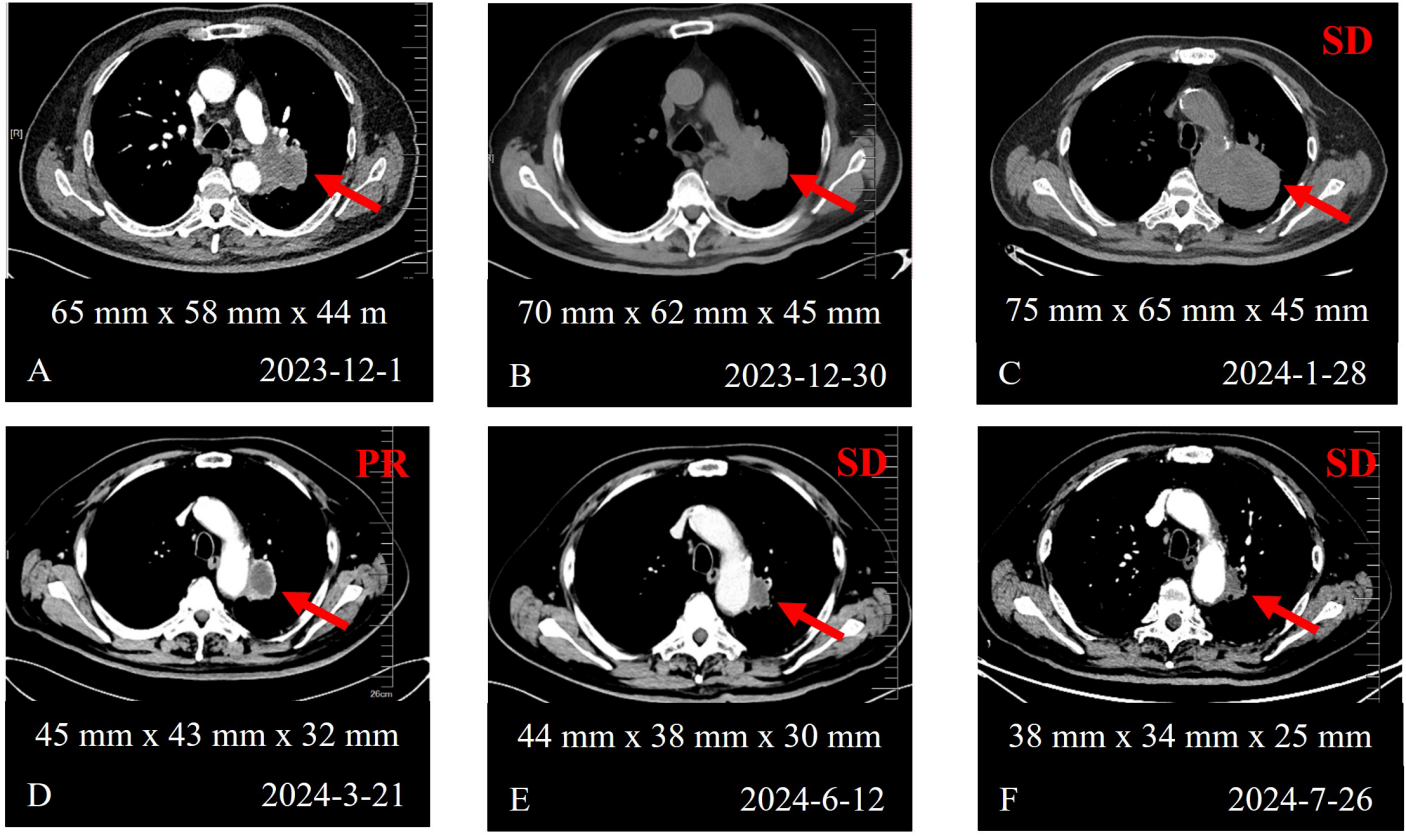
Chest CT images demonstrating the evolution of the primary lung lesion before and after treatment. **(A)** Baseline scan at initial diagnosis. **(B)** Scan before treatment initiation after definitive diagnosis. **(C)** Scan after 1 cycle of toripalimab, pemetrexed, and carboplatin. **(D)** Scan after 1 cycle of toripalimab, pemetrexed, and carboplatin, followed by 2 subsequent cycles of sotorasib combination therapy. **(E)** Scan after 2 cycles of sotorasib plus toripalimab combined with pemetrexed and carboplatin, and 1 additional cycle of sotorasib plus toripalimab. **(F)** Scan after 2 cycles of sotorasib plus toripalimab therapy.

A 70-year-old male patient presented in November 2023 with a left pulmonary mass detected during a routine physical examination. Initial chest CT revealed a lesion measuring approximately 65 mm x 58 mm x 44 mm in the left lung, suggestive of primary lung cancer (**Figure 1A**). Three percutaneous lung biopsies demonstrated interstitial fibrous tissue with lymphoplasmacytic infiltration but no definitive malignant cells. Subsequently, PET/CT revealed a ring-shaped, heterogeneously hypermetabolic mass in the left lung. Multiple hypermetabolic lesions with bone destruction were detected in the left scapula, right sixth rib, and left iliac bone, along with additional soft-tissue nodules near the left scapular margin, bilateral paracolic gutters, and mesentery, indicating widespread metastasis. A percutaneous biopsy of the soft tissue surrounding the left scapula revealed invasive carcinoma. Immunohistochemical analysis was positive for CKpan and CK7, and negative for vimentin, TTF-1, Napsin A and CK20. The Ki-67 index was approximately 80%. The pathological diagnosis was moderately differentiated invasive adenocarcinoma with mucin production. The patient was ultimately diagnosed with left lung adenocarcinoma and multiple metastases (cT3N3M1, stage IV). His ECOG performance status was 1, and body mass index (BMI) was 27.9 kg/m².

During the diagnostic period, the tumor enlargement (**Figure 1B**) and patient developed a fever peaking at 39.8 °C, accompanied by chills. All microbiological tests were negative, and anti-infective therapy was ineffective. Tumor-related fever was suspected and successfully controlled with oral naproxen. Given the high tumor burden and tumor-associated fever, empirical treatment with toripalimab (an anti-PD-1 antibody) in combination with pemetrexed and carboplatin was initiated for one cycle. Next-generation sequencing (NGS) was performed using a 551-gene panel at Simcere Diagnostic Laboratories. The identified mutations included *KRAS G12C* (27.77%), *TP53* p.R280I (35.67%), *RB1* c.1332 + 2T>C (54.96%), and *STK11* p.S216F (40.43%). *KEAP1* was wild-type and the tumor mutation burden (TMB) was 17.73 mutations/Mb. Programmed death-ligand 1 (PD-L1) expression was positive (TPS = 55%, CPS = 60).

After one cycle of chemoimmunotherapy, the patient’s fever worsened and his quality of life declined significantly. His fever necessitated oral dexamethasone (2.25 mg twice daily). A follow-up chest CT scan showed enlargement of the left lung lesion, which was designated as the target lesion (75 mm x 65 mm x 45 mm), and the disease was assessed as stable disease (SD) according to RECIST 1.1 (**Figure 1C**) (4). All subsequent response assessments were performed using RECIST 1.1 criteria. Given the rapid disease progression, aggressive tumor behavior, and the identification of a targetable *KRAS G12C* mutation, a multidisciplinary team (MDT) recommended a personalized treatment regimen consisting of sotorasib (720 mg once daily), toripalimab, pemetrexed and carboplatin. Sotorasib was subsequently escalated to the standard dose of 960 mg once daily after confirming acceptable tolerability. Following treatment initiation, the patient’s temperature decreased gradually, allowing for the eventual discontinuing of dexamethasone.

After three cycles, a partial response (PR) was achieved according to RECIST 1.1, with tumor shrinkage to 45 mm x 43 mm x 32 mm (**Figure 1D**). After five cycles, chemotherapy was discontinued due to grade 1 hepatotoxicity and grade 2 gastrointestinal adverse events, and the patient continued maintenance therapy with sotorasib plus toripalimab. Subsequent imaging showed SD, with the tumor measuring 44 mm x 38 mm x 30 mm (**Figure 1E**).

**Figure 2:**
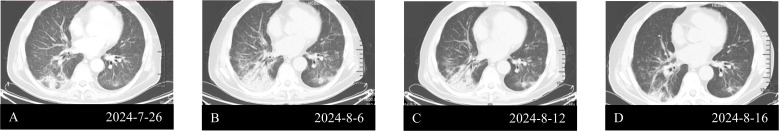
Chest CT images demonstrating the evolution of immune checkpoint inhibitor-related pneumonitis before and after treatment. **(A)** Baseline chest CT showing the initial extent of pneumonitis. **(B)** Scan after 12 days of anti-infective therapy. **(C)** Scan after 6 days of combination therapy with methylprednisolone (80 mg/day) and anti-infective treatment. **(D)** Scan after 4 days of methylprednisolone (60 mg/day) combined with anti-infective treatment.

In July 2024, the patient developed fever, productive cough, and dyspnea. Chest CT scan revealed pneumonia (**Figure 2A**), while the primary left lung lesion remained stable at 38 mm x 34 mm x 25 mm, with the response maintained as SD according to RECIST 1.1 (**Figure 1F**). NGS of bronchoalveolar lavage fluid identified opportunistic pathogens. Despite undergoing targeted anti-infective therapy, follow-up CT scan showed progression of pneumonia, predominantly with interstitial changes (**Figure 2B**), raising suspicion for immune checkpoint inhibitor (ICI)-related pneumonitis (CTCAE grade 2). Treatment with methylprednisolone led to gradual radiological improvement of pneumonitis (**Figures 2C, D**). Considering the patient’s overall condition, immunotherapy was discontinued, and sotorasib monotherapy was continued. The patient maintained SD, with no grade ≥3 adverse events.

**Figure 3:**
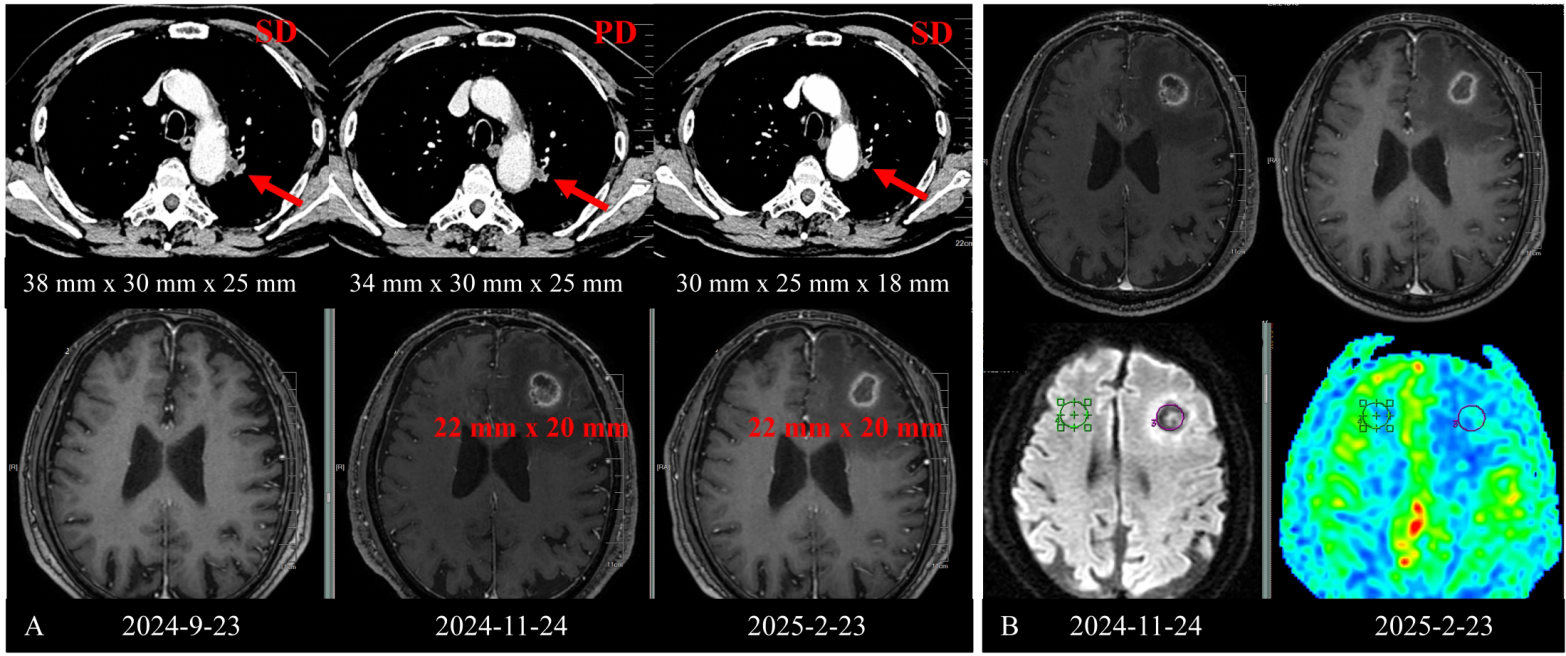
Scan demonstrating the evolution of the primary lung lesion and the left frontal lobe metastasis before and after treatment. **(A)** Scan showing the brain metastasis and the left lung lesion, including scans obtained before and after the brain metastasis identification. **(B)** Scan showing the lesion before and two months after radiotherapy.

In November 2024, brain MRI revealed a new metastatic lesion in the left frontal lobe (22 mm x 20 mm, **Figure 3A**) with significant peritumoral edema, and the disease was assessed as progression disease (PD) according to RECIST 1.1. At this point, the patient had achieved a PFS of approximately 10 months from treatment initiation, with progression manifesting as intracranial metastasis. Given the presence of a single new brain metastasis while extracranial disease remained stable, the patient was assessed as having oligoprogressive disease. Therefore, oral sotorasib therapy was continued, and the patient received local stereotactic radiotherapy (SRT) targeting the brain lesion, which was administered at a total dose of 47 Gy delivered in 10 fractions.

At the follow-up in February 2025, an MRI scan showed that the brain metastatic lesion measured 22 mm × 20 mm, with persistent peripheral enhancement surrounding the treated lesion (**Figure 3B**). Based on the patient’s clinical history and imaging findings, grade 0 (asymptomatic) radiation necrosis was suspected. Due to the large size of the lesion and extensive surrounding edema, bevacizumab (5 mg/kg every 14 days) was administered for six cycles to mitigate cerebral edema. Re-evaluation after four cycles confirmed SD.

**Figure 4:**
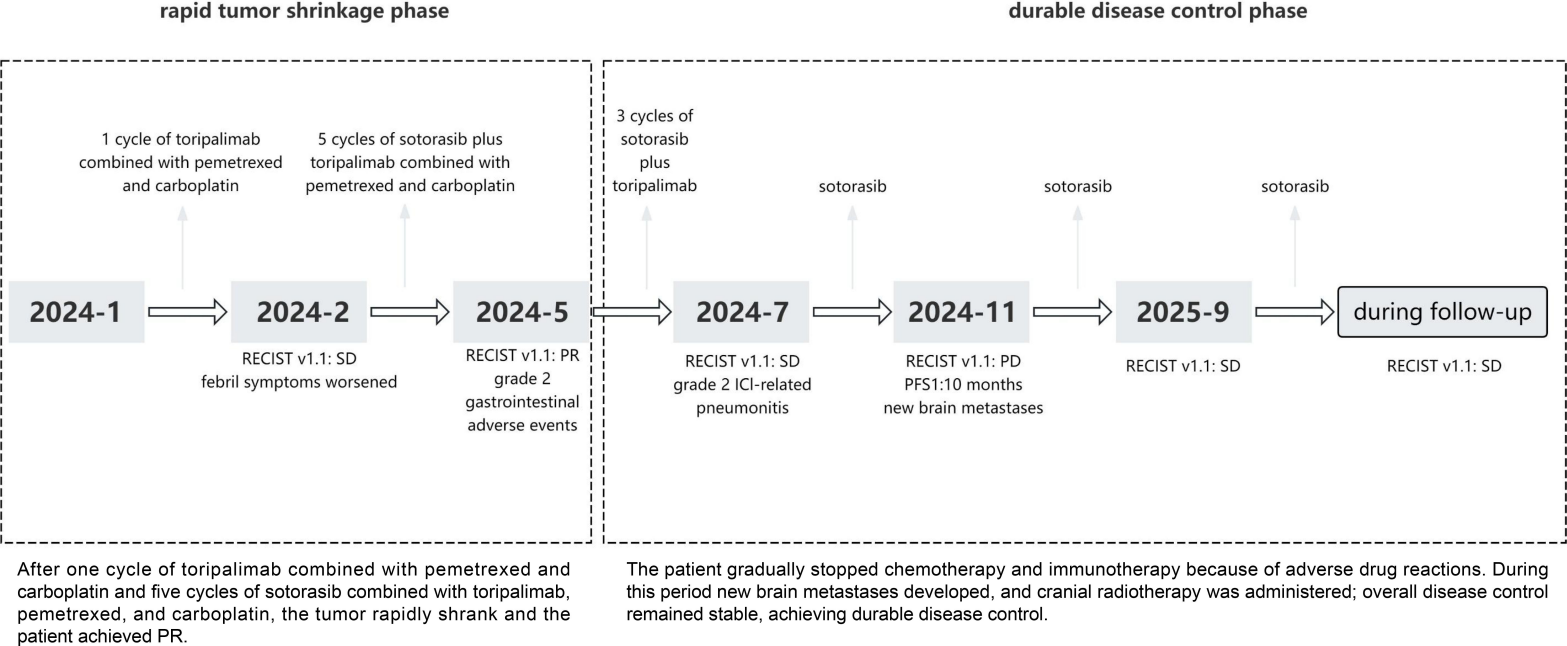
Treatment process.

In June 2025, the patient experienced reduced speaking and personality changes. A repeat brain MRI scan revealed grade 1 radiation necrosis. Bevacizumab (5 mg/kg every 14 days) was reinitiated in combination with methylprednisolone (40 mg once daily). The patient’s neurological symptoms improved significantly and the dosage of methylprednisolone was gradually reduced. At the latest follow-up, the patient remains on maintenance sotorasib therapy. Despite the development of brain metastasis during treatment, the intracranial lesion was well controlled through local radiotherapy, while extracranial disease remained stable. The overall clinical benefit duration was 23 months from the initiation of treatment (**Figure 4**).

## Discussion

Lung cancer remains the leading cause of cancer-related incidence and mortality in China and worldwide, with a 5-year overall survival rate of less than 20% (5–7). NSCLC are the most prevalent subtype, and *KRAS* mutations represent key oncogenic drivers (8). *KRAS* mutations drive the accumulation of oncogenic signaling and contribute to an immunosuppressive tumor microenvironment by inhibiting T-cell infiltration and recruiting immunosuppressive cells, consequently facilitating immune evasion and reducing the immunotherapy efficacy (9, 10). Conversely, *KRAS* mutations may also enhance immunotherapy by increasing TMB and tumor immunogenicity (11, 12).

More than 50% of *KRAS*-mutant NSCLC cases harbor co-mutations, most frequently involving *TP53*, *STK11*, and Kelch-like ECH-associated protein 1 (*KEAP1*), which can influence both treatment response and prognosis (13). *KRAS*/*TP53* co-mutations are generally associated with higher objective response rates (ORR) and improved outcomes following immunotherapy (14). Conversely, *KRAS*/*STK11* co-mutations give rise to an immunosuppressive tumor microenvironment, resulting in reduced responsiveness to immune checkpoint inhibitors (15).

*KRAS G12C* mutation account for approximately 40% of all *KRAS* mutations in lung adenocarcinoma and about 13% of all lung adenocarcinoma cases (16, 17). Studies have revealed that the *KRAS G12C* mutation is associated with significantly higher PD-L1 levels and higher TMB compared with other types and these microenvironment signatures may enhance the sensitivity of *KRAS G12C*-mutant patients to immune checkpoint inhibitors (18, 19). Moreover, the development of *KRAS G12C* inhibitors has substantially improved the prognosis of patients with *KRAS*-mutant NSCLC. However, the efficacy of *KRAS G12C* inhibitor monotherapy remains limited due to primary and acquired resistance mechanisms, including co-occurring *KEAP1* mutations, secondary *KRAS* mutations (e.g., R68S, H95D), pathway reactivation, and *KRAS G12C* amplification, leading to a median PFS of only 5–6 months (20, 21). To overcome these limitations, combination therapy strategies are being actively explored. The SCARLET study reported a median PFS of 6.6 months and a median overall survival (OS) of 20.6 months in patients with *KRAS G12C*-mutant non-squamous NSCLC, with treatment of sotorasib plus pemetrexed and platinum, either after prior immunotherapy or in the first-line setting (22). In the CodeBreaK 101 study, first-line treatment with sotorasib combined with pemetrexed and carboplatin achieved a median PFS of 10.8 months (23).

In this report, we describe an elderly patient with advanced lung adenocarcinoma harboring a *KRAS G12C* mutation co-occurring with *TP53*, *RB1*, and *STK11* mutations, along with high PD-L1 expression. The patient presented with a high tumor burden, rapid tumor progression, and tumor-related fever. Prior to the availability of molecular results, he received one cycle of toripalimab plus pemetrexed and carboplatin, which resulted in worsening of febrile symptoms and a poor clinical response, suggesting limited benefit from chemoimmunotherapy. Subsequently, NGS revealed a *KRAS G12C* mutation harboring concurrent mutations in *TP53*, *RB1*, and *STK11*. According to the NCCN guidelines, immunotherapy, either alone or in combination with chemotherapy, is recommended as the standard first-line treatment for newly diagnosed advanced NSCLC with *KRAS G12C* mutations (24). After one cycle of chemoimmunotherapy, radiographic evaluation showed SD according to RECIST 1.1, but the patient experienced clinical deterioration with worsening fever. Meanwhile, histopathological examination revealed mucin-producing *KRAS G12C*-mutant adenocarcinoma, a subtype known to respond poorly to chemotherapy and immunotherapy. Previous studies have indicated that patients with *KRAS*-mutant NSCLC derive only modest benefit from chemotherapy alone, that *STK11* co-mutation confers primary resistance to immune checkpoint inhibitors, and that monotherapy with a *KRAS G12C* inhibitor is prone to the development of resistance (25–28). Although sotorasib is currently approved only for subsequent-line treatment after progression on first-line therapy, delaying its administration until later line was considered to carry the risk of losing a therapeutic opportunity due to rapid tumor progression. Given the patient’s complex molecular profile and the aggressive nature of the disease, the case was discussed by MDT. The patient initially expressed concerns about the quadruple regimen and the potential risks and benefits of the combined treatment were fully explained to the patient. After obtaining informed consent, a personalized quadruple regimen comprising sotorasib, toripalimab, pemetrexed, and carboplatin was initiated as an investigational first-line therapy. After five cycles, the patient achieved a best response of PR, with resolution of fever and marked improvement in quality of life. The patient experienced rapid tumor shrinkage and achieved a PR, which was defined as the early rapid tumor shrinkage phase.

During treatment, the patient developed hepatotoxicity, gastrointestinal adverse events, and ICI-related pneumonitis, all of which were manageable with appropriate interventions. Subsequently, the patient developed a single brain metastasis, which was assessed as oligoprogressive disease. Oligoprogression is characterized by localized progression of a few metastatic lesions while the disease in other sites remains controlled or stable, with most studies adopting a cutoff of 3 to 5 progressive lesions (29). According to ASCO, ESMO, and NCCN guidelines, surgical resection is recommended for symptomatic lesions larger than 3 cm, whereas stereotactic radiosurgery (SRS) is preferred for smaller, asymptomatic metastases (30). Real-world evidence also indicates that SRS provides a superior one-year local brain control compared with surgical resection (31). Accordingly, SRS was administered for local intracranial control, while oral sotorasib was continued for systemic therapy. Regular brain MRI surveillance enabled early detection of radiation necrosis, which improved following combination therapy with bevacizumab and methylprednisolone. The best overall response was sustained extracranial disease control with intracranial oligoprogression during the subsequent immunotherapy and targeted therapy. The patient continued on maintenance sotorasib monotherapy, sustaining clinical benefit. At the most recent follow-up, the patient has maintained SD with a durable clinical benefit of 23 months. Owing to adverse drug reactions, the patient was switched to sotorasib monotherapy. However, new brain metastases developed during sotorasib monotherapy, indicating inadequate disease control. The patient then received cranial radiotherapy while continuing oral sotorasib, and subsequent follow-up assessments demonstrated SD. This period was defined as the durable disease control phase.

The patient harbored *KRAS G12C* mutation combined with co-mutations of *TP53*, *RB1*, and *STK11*, and high expression of PD-L1. *KRAS G12C* inhibitors can modulate the function of immune cells by reprogramming the tumor microenvironment. For instance, sotorasib activates adaptive anti-tumor immunity by reducing tumor-infiltrating immunosuppressive cells and enhancing infiltration and activity of antigen-presenting cells and CD8^+^ T cells (32). Studies have shown that the co-mutation of *KRAS G12C* combined with *TP53* correlates with an ‘immune-hot’ phenotype, whereas co-mutation with *STK11* is associated with an ‘immune-cold’ phenotype. Both of these can lead to a ‘heterogeneous immune phenotype’ even with the same *KRAS G12C* background (33). Although this patient harbored an *STK11* co-mutation, the observed high PD-L1 expression appeared to differ from the typical ‘immune-cold’ phenotype. This reflects the potential immunological heterogeneity in patients harboring concurrent *KRAS G12C* and *STK11* mutations and could possibly contribute to understanding the patient’s initial benefit from immunotherapy. Subsequently, immunotherapy and chemotherapy were discontinued due to adverse reactions. The patient continued on maintenance sotorasib monotherapy.

This case report has several limitations. Although the outcome was encouraging, the conclusions are constrained by the single-case nature of the report, tumor heterogeneity, interindividual variability, the off-label use of sotorasib and the limited clinical experience with the combination of sotorasib and chemoimmunotherapy, which restricts the generalizability of this treatment strategy. Therefore, this treatment approach should be regarded as investigational. Further phase III clinical trials and additional clinical experience are warranted to validate and to more clearly define the efficacy and safety of sotorasib combined with chemoimmunotherapy in patients with non-small cell lung cancer harboring *KRAS G12C* mutations.

## Conclusion

In summary, this case demonstrates the potential for achieving long-term clinical benefit through precision-targeted therapy in patients with advanced NSCLC harboring KRAS G12C mutations. An exploratory first-line sotorasib-based combination regimen could be considered a potential therapeutic option for this population. However, given the inherent limitations of a single case report in evaluating treatment efficacy and safety, this treatment strategy should be considered hypothesis-generating, and its clinical applicability needs to be validated in larger clinical studies.

## Data Availability

The original contributions presented in the study are included in the article/supplementary material. Further inquiries can be directed to the corresponding author.

## References

[1] Addeo A Banna GL Friedlaender A . Kras g12c mutations in NSCLC: from target to resistance. Cancers (Basel). (2021) 13:2541. doi: 10.3390/cancers13112541. PMID: 34064232 PMC8196854

[2] Zhang J Zhang J Liu Q Fan X-X Leung E-H Yao X-J . Resistance looms for KRAS G12C inhibitors and rational tackling strategies. Pharmacol Ther. (2022) 229:108050. doi: 10.1016/j.pharmthera.2021.108050. PMID: 34864132

[3] Tan AC Tan DSW . Targeted therapies for lung cancer patients with oncogenic driver molecular alterations. J Clin Oncol. (2022) 40:611–25. doi: 10.1200/JCO.21.01626. PMID: 34985916

[4] Eisenhauer EA Therasse P Bogaerts J Schwartz LH Sargent D Ford R . New response evaluation criteria in solid tumours: revised RECIST guideline (version 1.1). Eur J Cancer. (2009) 45:228–47. doi: 10.1016/j.ejca.2008.10.026. PMID: 19097774

[5] Han B Zheng R Zeng H Wang S Sun K Chen R . Cancer incidence and mortality in China, 2022. J Natl Cancer Cent. (2024) 4:47–53. doi: 10.1016/j.jncc.2024.01.006. PMID: 39036382 PMC11256708

[6] Bray F Laversanne M Sung H Ferlay J Siegel RL Soerjomataram I . Global cancer statistics 2022: GLOBOCAN estimates of incidence and mortality worldwide for 36 cancers in 185 countries. CA Cancer J Clin. (2024) 74:229–63. doi: 10.3322/caac.21834. PMID: 38572751

[7] Allemani C Matsuda T Di Carlo V Harewood R Matz M Nikšić M . Global surveillance of trends in cancer survival 2000–14 (CONCORD-3): analysis of individual records for 37 513 025 patients diagnosed with one of 18 cancers from 322 population-based registries in 71 countries. Lancet. (2018) 391:1023–75. doi: 10.1016/S0140-6736(17)33326-3. PMID: 29395269 PMC5879496

[8] Zhang Y Vaccarella S Morgan E Li M Etxeberria J Chokunonga E . Global variations in lung cancer incidence by histological subtype in 2020: a population-based study. Lancet Oncol. (2023) 24:1206–18. doi: 10.1016/S1470-2045(23)00444-8. PMID: 37837979

[9] Rosell R Codony-Servat J González J Santarpia M Jain A Shivamallu C . Kras g12c-mutant driven non-small cell lung cancer (NSCLC). Crit Rev Oncol Hematol. (2024) 195:104228. doi: 10.1016/j.critrevonc.2023.104228. PMID: 38072173

[10] Xu M Zhao X Wen T Qu X . Unveiling the role of KRAS in tumor immune microenvironment. BioMed Pharmacother. (2024) 171:116058. doi: 10.1016/j.biopha.2023.116058. PMID: 38171240

[11] Ghazali N Garassino MC Leighl NB Bestvina CM . Immunotherapy in advanced, KRAS G12C-mutant non-small-cell lung cancer: current strategies and future directions. Ther Adv Med Oncol. (2025) 17:17588359251323985. doi: 10.1177/17588359251323985. PMID: 40093982 PMC11907553

[12] Lui K Cheung K-K Ng W-M Wang Y Au DWH Cho WC . The impact of genetic mutations on the efficacy of immunotherapies in lung cancer. Int J Mol Sci. (2024) 25:11954. doi: 10.3390/ijms252211954. PMID: 39596025 PMC11594099

[13] Chen H Huang D Lin G Yang X Zhuo M Chi Y . The prevalence and real-world therapeutic analysis of Chinese patients with KRAS-Mutant Non-Small Cell lung cancer. Cancer Med. (2022) 11:3581–92. doi: 10.1002/cam4.4739. PMID: 35394121 PMC9554448

[14] Budczies J Romanovsky E Kirchner M Neumann O Blasi M Schnorbach J . Kras and TP53 co-mutation predicts benefit of immune checkpoint blockade in lung adenocarcinoma. Br J Cancer. (2024) 131:524–33. doi: 10.1038/s41416-024-02746-z. PMID: 38866964 PMC11300455

[15] Knetki-Wróblewska M Wojas-Krawczyk K Krawczyk P Krzakowski M . Emerging insights into STK11, KEAP1 and KRAS mutations: implications for immunotherapy in patients with advanced non-small cell lung cancer. Transl Lung Cancer Res. (2024) 13:3718–30. doi: 10.21037/tlcr-24-552. PMID: 39830769 PMC11736579

[16] Yang H Zhu A Niu Y Ma W Xu K Jia Y . Distinct molecular subtypes of KRASG12C‐mutant lung adenocarcinoma: Insights into clinical outcomes, tumour microenvironments and therapeutic strategies. Clin Transl Med. (2025) 15:e70490. doi: 10.1002/ctm2.70490. PMID: 41025426 PMC12481212

[17] Veluswamy R Mack PC Houldsworth J Elkhouly E Hirsch FR . Kras g12c–mutant non–small cell lung cancer. J Mol Diagn. (2021) 23:507–20. doi: 10.1016/j.jmoldx.2021.02.002. PMID: 33618059

[18] Arbour KC Rizvi H Plodkowski AJ Hellmann MD Knezevic A Heller G . Treatment outcomes and clinical characteristics of patients with KRAS-G12C–mutant non–small cell lung cancer. Clin Cancer Res. (2021) 27:2209–15. doi: 10.1158/1078-0432.CCR-20-4023. PMID: 33558425 PMC8771577

[19] Sebastian M Eberhardt WEE Hoffknecht P Metzenmacher M Wehler T Kokowski K . Kras g12c-mutated advanced non-small cell lung cancer: A real-world cohort from the German prospective, observational, nation-wide CRISP Registry (AIO-TRK-0315). Lung Cancer. (2021) 154:51–61. doi: 10.1016/j.lungcan.2021.02.005. PMID: 33611226

[20] Dy GK Govindan R Velcheti V Falchook GS Italiano A Wolf J . Long-term outcomes and molecular correlates of sotorasib efficacy in patients with pretreated KRAS G12C-mutated non-small-cell lung cancer: 2-year analysis of CodeBreaK 100. J Clin Oncol. (2023) 41:3311–7. doi: 10.1200/JCO.22.02524. PMID: 37098232 PMC10414711

[21] de Langen AJ Johnson ML Mazieres J Dingemans AC Mountzios G Pless M . Sotorasib versus docetaxel for previously treated non-small-cell lung cancer with KRAS(G12C) mutation: a randomised, open-label, phase 3 trial. Lancet. (2023) 401:733–46. doi: 10.1016/S0140-6736(23)00221-0. PMID: 36764316

[22] Akamatsu H Sakata S Azuma K Yoshioka H Uemura T Tsuchiya-Kawano Y . A single-arm phase 2 study of sotorasib plus carboplatin and pemetrexed in patients with advanced nonsquamous NSCLC with KRAS G12C mutation (WJOG14821L, SCARLET). J Thorac Oncol. (2025) 20:775–85. doi: 10.1016/j.jtho.2025.01.006. PMID: 39828218

[23] Li BT Clarke JM Felip E Ruffinelli JC Garrido P Zugazagoitia J . Sotorasib plus carboplatin and pemetrexed in KRAS G12C advanced NSCLC: Updated analysis from the international CodeBreaK 101 trial. J Clin Oncol. (2024) 42:8512. doi: 10.1200/JCO.2024.42.16_suppl.8512. PMID: 41735675

[24] Skoulidis F Li BT Dy GK Price TJ Falchook GS Wolf J . Sotorasib for lung cancers with KRAS p.G12C mutation. N Engl J Med. (2021) 384:2371–81. doi: 10.1056/NEJMoa2103695. PMID: 34096690 PMC9116274

[25] Julian C Pal N Gershon A Evangelista M Purkey H Lambert P . Overall survival in patients with advanced non-small cell lung cancer with KRAS G12C mutation with or without STK11 and/or KEAP1 mutations in a real-world setting. BMC Cancer. (2023) 23:352. doi: 10.1186/s12885-023-10778-6. PMID: 37069542 PMC10108521

[26] Awad MM Liu S Rybkin II Arbour KC Dilly J Zhu VW . Acquired resistance to KRAS G12C inhibition in cancer. N Engl J Med. (2021) 384:2382–93. doi: 10.1056/NEJMoa2105281. PMID: 34161704 PMC8864540

[27] Di Federico A Hong L Elkrief A Thummalapalli R Cooper AJ Ricciuti B . Lung adenocarcinomas with mucinous histology: clinical, genomic, and immune microenvironment characterization and outcomes to immunotherapy-based treatments and KRAS(G12C) inhibitors. Ann Oncol. (2025) 36:297–308. doi: 10.1016/j.annonc.2024.11.014. PMID: 39637943 PMC11845285

[28] Giaccone G He Y . Current knowledge of small cell lung cancer transformation from non-small cell lung cancer. Semin Cancer Biol. (2023) 94:1–10. doi: 10.1016/j.semcancer.2023.05.006. PMID: 37244438

[29] Tsai CJ Yang JT Shaverdian N Patel J Shepherd AF Eng J . Standard-of-care systemic therapy with or without stereotactic body radiotherapy in patients with oligoprogressive breast cancer or non-small-cell lung cancer (Consolidative Use of Radiotherapy to Block [CURB] oligoprogression): an open-label, randomised, controlled, phase 2 study. Lancet. (2024) 403:171–82. doi: 10.1016/S0140-6736(23)01857-3. PMID: 38104577 PMC10880046

[30] Okuno T Isobe T Tsubata Y . Current pharmacologic treatment of brain metastasis in non-small cell lung cancer. Clin Exp Metastasis. (2024) 41:549–65. doi: 10.1007/s10585-024-10276-4. PMID: 38466521 PMC11499348

[31] Rostampour N Badrigilan S Rezaeian S Sarbakhsh P Meola A Choupani J . Efficacy of stereotactic radiosurgery as single or combined therapy for brain metastasis: a systematic review and meta-analysis. Crit Rev Oncol Hematol. (2023) 186:104015. doi: 10.1016/j.critrevonc.2023.104015. PMID: 37146702

[32] Zhang F Wang B Wu M Zhang L Ji M . Current status of KRAS G12C inhibitors in NSCLC and the potential for combination with anti-PD-(L)1 therapy: a systematic review. Front Immunol. (2025) 16:1509173. doi: 10.3389/fimmu.2025.1509173. PMID: 40303413 PMC12037499

[33] Qiao M Zhou F Liu X Jiang T Wang H Li X . Targeting focal adhesion kinase boosts immune response in KRAS/LKB1 co-mutated lung adenocarcinoma via remodeling the tumor microenvironment. Exp Hematol Oncol. (2024) 13:11. doi: 10.1186/s40164-023-00471-6. PMID: 38291516 PMC10826079

